# UTILIZING PALLIATIVE LEADERS IN FACILITIES TO TRANSFORM CARE: THE UPLIFT CLINICAL TRIAL IMPLEMENTATION

**DOI:** 10.1093/geroni/igad104.1809

**Published:** 2023-12-21

**Authors:** Kathleen Unroe, John Cagle, Mary Ersek

**Affiliations:** Indiana University, Indianapolis, Indiana, United States; University of Maryland, Baltimore, Baltimore, Maryland, United States; University of Pennsylvania, Philadelphia, Pennsylvania, United States

## Abstract

UPLIFT is an NIH funded clinical trial with a goal of improving quality of care for people with moderate to advanced dementia in nursing homes. The UPLIFT intervention consists of specialized training in palliative care and dementia care for two nursing facility staff who are identified as Palliative Care Leads. All staff training on the fundamentals of palliative care is also delivered. In addition to this internal capacity building, UPLIFT integrates specialty medical palliative care consultants into the care team. Palliative Care Leads screen residents for palliative care needs which prompt clinical consultations. Outcomes include surrogate (both nursing facility staff and family) reported symptoms of eligible residents. A total of 16 nursing facilities are enrolled in this stepped wedge trial, which concludes in 2025. Numerous implementation challenges have been identified and navigated throughout the trial. These include Palliative Care Lead turnover, nursing facility leadership turnover, communication with primary medical teams, and integration of palliative care NPs and MDs into the clinical care of the residents. Data on challenges is captured on regular fidelity check-ins with nursing home staff and interviews with Leads and palliative care providers. A description of these challenges and strategies the team is using to navigate them will be presented, including adaptations to study design and the intervention.

